# How I treat Parkinson's disease

**DOI:** 10.1590/0004-282X-ANP-2022-S126

**Published:** 2022-08-12

**Authors:** Egberto Reis Barbosa, João Carlos Papaterra Limongi, Hsin Fen Chien, Pedro Melo Barbosa, Marcela Reuter Carréra Torres

**Affiliations:** 1 Universidade de São Paulo, Faculdade de Medicina, Hospital das Clínicas, Divisão de Neurologia, Grupo de Distúrbios do Movimento, São Paulo, SP, Brazil.

**Keywords:** Parkinson Disease, Therapeutics, Levodopa, Doença de Parkinson; Terapêutica, Levodopa

## Abstract

**Background:**

Parkinson’s disease (PD) is a complex neurodegenerative condition. Treatment strategies through all stages of disease progression could affect quality of life and influence the development of future complications, making it crucial for the clinician to be on top of the literature.

**Objective:**

This paper reviews the current treatment of PD, from early to advanced stages.

**Methods:**

A literature review was conducted focusing on the treatment of PD, in the different stages of progression.

**Results:**

Every individual with a new diagnosis of PD should be encouraged to start exercising regularly. In the early stage, treatment should focus on using the lowest dose of levodopa or combination therapy that provides maximum functional capacity, and does not increase the risk of complications, such as peak dose dyskinesias and impulse control disorders. At the moderate and advanced stages, motor fluctuations and complications of treatment dominate the picture, making quality of life one important issue. Rehabilitation programs can improve motor symptoms and should be offered to all patients at any stage of disease progression.

**Conclusion:**

Many factors need to be considered when deciding on the best treatment strategy for PD, such as disease progression, presence of risk factors for motor and behavioral complications, potential side effects from dopaminergic therapy and phenotypical variabilities. Treatment should focus on functional capacity and quality of life throughout the whole disease course.

## RESUMO

**Antecedentes:**

A doença de Parkinson (DP) é uma doença neurodegenerativa complexa. As estratégias de tratamento ao longo de todos os estágios da evolução podem influenciar a qualidade de vida e o desenvolvimento de futuras complicações e, portanto, devem ser devidamente conhecidas pelos médicos que assistem o paciente.

**Objetivo:**

Neste artigo são revistos os tratamentos atuais para a DP, desde o estágio inicial até os estágios avançados.

**Métodos:**

Uma revisão da literatura foi realizada com enfoque em diferentes estágios da doença.

**Resultados:**

Todos os pacientes que recebem o diagnóstico de DP devem ser orientados a praticar atividades físicas regularmente. Em fase inicial do tratamento a estratégia consiste em usar doses mínimas de levodopa ou terapias combinadas que propiciem máxima capacidade funcional, que não aumentem o risco de complicações motoras, tais como discinesias induzidas por levodopa, ou não motoras, como transtornos do controle do impulso. Em estágio moderado ou avançado da doença, complicações como flutuações motoras e discinesias tornam-se relevantes e devem ser manejadas adequadamente. Programas de reabilitação devem ser oferecidos para os pacientes em todos os estágios da DP.

**Conclusões:**

Vários fatores devem ser considerados ao se escolher a melhor estratégia para o tratamento da DP. Entre eles destacam-se: fatores de risco para o desenvolvimento de complicações motoras e comportamentais, potenciais efeitos colaterais da terapia dopaminérgica e variabilidade fenotípica. O objetivo das estratégias de tratamento ao longo de toda a evolução da doença é a melhoria da capacidade funcional e da qualidade de vida dos pacientes.

## INTRODUCTION

Parkinson’s disease (PD) is the second most common neurodegenerative disorder worldwide. PD prevalence ranges from 102 to 190 per 100,000 in Western countries^1^, affecting both men and women, but being slightly more common in men. The disease typically develops after the age of fifty and may affect up to 2% of the population older than 65 years old^2^. From 1900 to 2015 the projection of the number of people with PD doubled to over 6 million, mainly due to ageing population. The number of people affected is projected to a staggering 12.9 million people affected by 2,040^3^. Early-onset PD denotes disease onset below age 40, and juvenile PD when age of disease onset is below 21 years.

PD is a complex disorder with a broad spectrum of motor and non-motor features that require an individualized therapeutic approach. Several guidelines for the treatment of PD are available in the literature^4^. The main drugs used for the treatment of motor manifestations of the disease are: levodopa, dopamine agonists, catechol-o-methyltransferase inhibitors, monoamine oxidase inhibitors, amantadine and anticholinergics^5^. 

## LEVODOPA

Levodopa is the most effective drug for the treatment of symptoms of Parkinson disease. In the 1970s, it was discovered that adding a dopa decarboxylase inhibitor to levodopa to reduce its peripheral metabolism would lessen treatment side effects and show better symptom control. Levodopa treatment contributes to greater activity levels, independence, employability and consequently improved patient’s quality of life^6^. Levodopa dose should be kept as low as possible to maintain good function in order to reduce the risk of motor complications.

For patients with prolonged off periods or severe dysphagia an encapsulated levodopa powder can be administered orally via an inhaler, but this preparation is not available in our country^7^. 

Levodopa may lead to the development of longterm motor complications, including abnormal involuntary movements such as dyskinesias, dystonia, and response fluctuations. 

The pathogenesis of motor complications associated with chronic levodopa therapy is still not fully understood. Theories include: presynaptic neuronal degeneration leading to insufficient buffering of released levodopa; postsynaptic changes in dopamine receptor number and its sensitivity, partially caused by presynaptic changes; and pharmacokinetic and pharmacodynamics changes of chronic dopaminergic therapy. Motor complications may be also related to the pulsatile use of levodopa, which leads to an imbalance of basal ganglia opioid concentrations and resetting of voltage gated channels in N-methyl-D-aspartate (NMDA) receptors^8^ ^,^ ^9^.

Different levodopa formulations have been developed to provide more desirable delivery to avoid or prevent levodopa-related complications, but most of them are ineffective for these goals. A continuous intrajejunal infusion of levodopa-carbidopa intestinal gel has been found effective in smoothing out motor fluctuations^5^.

## DOPAMINE AGONISTS

Dopamine agonists (DA) act directly stimulating dopamine brain receptors in There are two DA subclasses: ergoline and non-ergoline agonists. Ergoline dopamine agonists include bromocriptine, pergolide, lisuride, and cabergoline, whereas ropinirole, pramipexole and rotigotine (patch application) are non-ergoline agonists. Apomorphine was the first DA shown to improve parkinsonian symptoms, but it has to be administered subcutaneously (not available in our country). Only non-ergot dopamine agonists, such as pramipexole and rotigotine, both available in Brazil, should be used because ergot agonists may cause pulmonary and cardiac fibrosis.

DA has been used as monotherapy in de novo patients with the intention of delaying levodopa treatment. DA are also used as adjunct to levodopa treatment in patients exhibiting motor fluctuation. DA rather than levodopa use appears to postpone onset of motor complications and dyskinesias, since motor complications may be related to the pulsatile use of levodopa as previously mentioned. DA display longer half-lives and differences in receptor selectivity, while dopaminergic therapy may lead to the development of Impulse control disorders, more common in patients on dopamine agonists therapy.

For patients with severe off periods and delayed onset with subsequent dosing, self-administered subcutaneous apomorphine injections (available outside Brazil) can be used to obtain a faster motor response. 

## CATECHOL-O-METHYLTRANSFERASE INHIBITORS (COMTI)

When peripheral decarboxylation is blocked by carbidopa or benserazide, the next n metabolic pathway of levodopa is O-methylation by COMT. COMTIs block dopamine degrading enzymes, prolonging the benefits of levodopa by improving its bioavailability and inhibiting the formation of 3-O-methyldopa. 

Entacapone (the only COMTI available in Brazil) inhibits COMT peripherally and tolcapone inhibits it both centrally and peripherally. Opicapone is a new generation once-daily administration COMTI, not yet available in Brazil. These drugs prolong the antiparkinsonian effect of levodopa and also allow levodopa dose reduction. Combination of COMT inhibitors and levodopa is indicated in patients with wearing off.

## MONOAMINE OXIDASE INHIBITORS (MAOI)

Most of the MAO in the brain are type B and play an important role in the monoamine breakdown, such as dopamine. Three MAO inhibitors are available for the treatment of PD: selegiline, rasagiline and safinamide.

Although selegiline and rasagiline are most frequently used in early and mild PD, these MAOI are also effective in patients with moderately advanced PD with levodopa-related motor complications. A third generation of MAOI, safinamide, administered once daily (50-100 mg/day), has been found to increase patients‘ on time without troublesome dyskinesia and to reduce daily and morning off times^10^. Safinamide is a reversible MAOI, it also reduces neuronal dopamine reuptake and blocks voltage-dependent activated sodium channel and intracellular calcium entry, reducing neuronal glutamate release. 

## AMANTADINE

Amantadine, originally used as an antiviral drug, was fortituitously recognized to be a treatment for PD despite being a weak therapeutic agent. This drug increases dopamine release and blocks dopamine reuptake. Recent studies have demonstrated that amantadine is a weak NMDA glutamate receptor antagonist. Thus, amantadine can influence glutamatergic neurotransmission in corticostriatal synapses or at the subthalamic-internal pallidal synaptic level. Moreover, recent randomized clinical trials have also shown that amantadine reduces dyskinesias and motor fluctuations in patients receiving levodopa^11^. Immediate-release amantadine is used off label for dyskinesia. Extended-release amantadine preparations was approved by the FDA for dyskinesia^12^.

## ANTICHOLINERGICS

The oldest class of medicines to treat PD is the anticholinergic (ACH) drugs. Biperiden and trihexyphenidyl reduce cholinergic activity contributing to reestablish the balance between striatal cholinergic and dopaminergic activity. The most recognized ACH drug use is to treat tremor in early or young onset PD, but ACH drugs do not significantly affect bradykinesia or rigidity. The most common side effects of ACH include dry mouth, blurred vision, constipation, and difficulty voiding the bladder. ACH can cause confusional states in elderly patients. 

## SURGICAL TREATMENT

Deep brain stimulation (DBS) of the subthalamic nucleus or internal globus pallidus are indicated to manage patients with severe dyskinesias and motor fluctuations. In the topic “Treatment of the advanced phase of PD” additional brief comments about this subject will be made. Details about the indications criteria and complications related to this procedure will be discussed in another article of this issue. 

## MODIFYING-DISEASE THERAPIES

Multiple lines of research point to several pathways which may contribute to neurodegeneration in PD: mitochondrial dysfunction, defective autophagy, abnormal protein accumulation of α-synuclein and synaptic impairment. Although effective therapies are available for the symptomatic control of PD treatments to halt the progression of the neurodegeneration process do not exist so far. Development of disease-modifying therapies to slow, mitigate or prevent PD is crucial ,and new approaches to development of these agents have been proposed recently by Kieburtz et al.^13^.

## MANAGEMENT OF EARLY PD

Early stage PD begins when the first motor symptoms are noticed and the diagnosis is established. This stage may last for months or a few years, until symptoms begin to interfere with daily activities and quality of life is affected. 

There are many treatment options to control symptoms in early stages of PD. Optimal management in individual patients depends on a number of variables such as age, cognitive status, symptom severity, predominance of bradykinesia or tremor, degree of dominant hand involvement, functional disability and employment status, among others. Medical treatment of early PD consists of dopamine replacing strategies to relieve motor symptoms, but eventually, other medical interventions may be required to treat nonmotor symptoms, such as depression or bowel constipation. No neuroprotective therapy is currently available to treat PD. 

Four main classes of drugs are currently considered to be useful for initial PD: monoamine oxidase type B (MAO-B)) inhibitors, amantadine, dopamine agonists (DA) and levodopa. Occasionally, anticholinergic drugs may be used in young patients with predominant tremor. These drugs have different pharmacological profiles, potencies and side effects. None of them has been found to be neuroprotective. MAO-B inhibitors and amantadine are less poten,t and can be used in patients with mild symptoms. Side effects are usually mild and well tolerated. DAs have a more potent dopaminergic effect and a longer half-life than levodopa. There is a lower risk of producing motor complications compared to levodopa, but adverse effects can be troublesome and include somnolence, hallucinations and impulse control disorders. Levodopa is the most effective drug and all patients will use this drug at some point in the course of the disease. However, short half-life and higher risk of development of dyskinesias are limitations to be considered^14^ ^,^ ^15^.

Some clinicians have advocated that levodopa should be used as the initial therapy in spite of the known risk of producing motor fluctuations and dyskinesias^16^. The main argument is that there is no proof that levodopa is toxic to the brain or that the cause of motor complications is levodopa itself. In fact, there are reasons to indicate that dyskinesias correlate with disease severity and duration and not with duration of levodopa therapy. Moreover, it may be reasonable to argue that the most effective drug should be used in the early stages to provide the best quality of life from the start. Furthermore, the increasing recognition of severe impulse control disorders have cautioned against the use of DAs in more vulnerable patients^17^.

Moreover, those who prefer to delay the introduction of levodopa in favor of other dopamine-sparing drugs do not recommend postponing its use beyond the patient clinical requirement. 

Two pivotal studies deserve mention regarding early levodopa treatment. The ELLDOPA study was a controlled clinical trial aimed to evaluate the effect of levodopa on the clinical course of PD. Results showed that subjects treated with levodopa had less clinical progression after a two-week washout period compared to the placebo group, and that this effect was dose-dependent. In spite of some discordance between clinical results and ligand imaging findings suggesting increased degeneration in dopamine terminal neurons in the levodopa arms, the overall conclusion of this study was that levodopa is not toxic^18^. 

The other trial was the LEAP study, which was a delayed-start clinical trial to investigate whether early treatment with levodopa had a beneficial disease modifying effect on PD. The results showed no differences between groups and the authors concluded that levodopa does not have a disease modifying effect^19^.

A recent report of the American Academy of Neurology reviewed the current evidence on the options available for initiating treatment of motor symptoms in early-stage PD. The main recommendations are summarized below: (Pringsheim)^20^.

### Levodopa vs. DAs vs. MAO-B inhibitors

Clinical trials have failed to provide evidence of disease modifying effects when therapy is initiated with any class of drugs. Studies comparing treatment with levodopa with treatment with MAO-B inhibitors demonstrate greater improvement with levodopa and > 60% of individuals randomized to MAO-B inhibitors will require additional therapy within two to three years. (PD MED)^21^.

Initial treatment of early PD with levodopa provides greater benefit for motor symptoms, but is more likely to induce dyskinesias than initial treatment with DAs. Treatment with DAs may be more likely to cause more hallucinations compared with levodopa, but the difference is small for the first five years. Treatment with DAs in early PD carries a higher risk of impulse control disorders. Patient characteristics may provide clues to the risk of adverse effects. Younger age at onset, lower body weight, female gender and disease severity are predisposing factors for levodopa-induced dyskinesias. Predisposing factors for impulse control disorders are younger age, male gender and history of previous mood disorders. Cognitive and behavioral adverse effects are more common in older patients treated with DAs. 

Overall recommendations regarding comparison studies among drugs are as follows:

- In patients who seek treatment for motor symptoms, levodopa should be the initial dopaminergic therapy;
- DAs may be prescribed as initial drug in patients < 60 years, whio are at higher risk for dyskinesias;
- DAs should not be prescribed to patients older than 70 years, or with a history of impulse control disorders, cognitive impairment, daytime sleepiness or hallucinations.

### Recommendations for levodopa

Studies comparing immediate-release, extended release or levodopa/carbidopa/entacapone formulations did not detect significant differences. Recommendations for levodopa prescription are:

- Immediate-release should be preferred over extended release or association with entacapone formulations in patients with early PD;
- Lowest effective doses should be prescribed to minimize risk of adverse effects;
- Patients should be advised that higher dosages increase the risk of dyskinesias;
- Patients should be advised that taking levodopa with meals may decrease levodopa effect, butthis is usually not a problem in early disease stages.

Recommendations for DAs

- Patients and caregivers should be informed of side effects of Das, such as impulse control disorders, sudden-onset sleepiness, postural hypotension and hallucinations;
- Patients should be screened for the above symptoms and for cognitive impairment;
- There is no compelling evidence that pramipexole extended-release vs. pramipexole immediate-release was associated with more favorable UPDRS scores;
- There are preliminary reports that long-acting and transdermal formulations of DAs have lower rates of impulse control disorders than short-acting formulations;
- The lowest effective dose of DAs should be prescribed.

### Recommendations for MAO-B inhibitors

- Most patients on monotherapy with a MAO-B inhibitor will require additional therapy after two to three years. This class of drug is associated with higher risk of adverse effects on discontinuation compared to levodopa treatment;
- There are no studies comparing the efficacy of selegiline, rasagiline and safinamide;
- Both drugs are superior to placebo for the treatment of motor symptoms;
- Although serotonin syndrome is rarely reported in PD, prescribing information cautions against concomitant use of MAO-B inhibitors and serotonin reuptake inhibitors.

## TREATMENT OF ADVANCED PARKINSON’S DISEASE

### Definition of advanced PD and its challenges

Advanced stage PD is defined by the presence of functional limitation despite optimized treatment, and occurrence of motor complications associated with dopamine replacement therapy (DRT), such as motor fluctuations, dyskinesias and psychosis. Postural instability and/or regular falls, gait freezing, postural abnormalities (camptocormia, Pisa syndrome) and cognitive impairment are the main conditions associated with disability at this stage, negatively affecting quality of life of affected individuals. The term end stage PD is also used by some authors when there is significant disability and poor response to available treatments^22^. In the Hoehn and Yahr (H&Y) classification, advanced PD is classified as stages 4 or 5^23^.

In the advanced stage of the disease most patients present with symptoms that are not improved by levodopa. Hely and colleagues showed that after 15 years of disease progression the main motor symptoms affecting individuals with PD are: motor complications of dopamine replacement treatment (dyskinesias and wearing-off), regular falls and choking. The main nonmotor symptoms (NMS) are cognitive decline, depression, hallucinations, urinary incontinence and postural hypotension^24^. Periodic consultations with a movement disorders neurologist could help manage the complications of DRT and guide treatment decisions, particularly concerning advanced therapies, such as infusion therapies and surgical procedures^25^.

### Non-pharmacological treatment of advanced PD

Physical therapy and exercise can improve PD symptoms not usually ameliorated by DRT, such as gait impairment and freezing, postural instability, falls, and non-motor symptoms, such as depression, constipation, apathy, and fatigue^26^. It is good practice to recommend physical exercise to all patients with a recent diagnosis of PD. Individuals with advanced PD, on the other hand, are at greater risk of injury from exercising and need a personalized approach. At this stage, physical therapy should focus on prevention of falls, improving transfers, posture, balance, and gait. This could be achieved by assisted active exercising and training of cognitive movement strategies. In patients with H&Y 5 physical therapy can help preventing contractures and pressure sores as well as help with postural adjustments^27^. An occupational therapist could contribute to caregiver education, helping to reduce caregiver burden, to improve quality of care, and to advise environmental adaptations to reduce risk of falls and injuries^28^.

Speech problems such as hypophonia and dysarthria frequently fail to improve with levodopa, and can worsen as the disease progresses, being a significant source of distress for individuals with PD, particularly in the advanced stage. Speech rehabilitation can improve dysarthria and possibly increase voice pitch, and should therefore be offered to any patient suffering from these symptoms^29^. Dysphagia is a common symptom in advanced PD3 and should prompt referral to a speech therapist.

### Pharmacological treatment of advanced PD

*Motor complications of dopaminergic treatment*

Motor fluctuations are the main complications of levodopa therapy in advanced PD. Motor fluctuations are predictable: end of dose (wearing-off), unpredictable: OFFs, ON-OFF phenomenon, delayed-on and dose failures. Troublesome motor fluctuations affect 43% of patients with PD after five years of disease progression^30^ ^,^ and become more frequent with time, affecting approximately 80% of individuals 10 years after diagnosis. Motor fluctuations are associated with younger age at disease onset, longer disease duration and higher doses of levodopa^31^ ^,^ ^32^. Unpredictable ON-OFF phenomenon usually occurs later in disease course^33^.

Dyskinesias are involuntary movements that occur in an individual with PD showing a temporal relationship to levodopa ingestion^34^. Dyskinesias are associated with longer disease duration, affecting 59% of individuals after 10 years of disease progression. Risk factors for development of levodopa-induced dyskinesias (LID) are younger age at PD onset, higher levodopa dose and presence of motor fluctuations^35^.

Different types of dyskinesias are seen in PD. Dyskinesias that occur at peak levodopa serum concentration are the most common, these dyskinesias are choreiform, appear initially in the more affected body side, and do significantly impact on quality of life.1 Dystonia is commonly painful, can affect as many as 30% of individuals with PD, and is seen in OFF periods^35^ ^,^ ^36^. Diphasic dyskinesias are rare, occur a few moments before levodopa kicks in and wears off, and are usually manifested as stereotyped movements of one or both legs^22^.

*Treatment of motor complications*

The likelihood of developing complications from dopamine replacement therapy is substantially increased in the advanced stage of PD. The treating physician faces complex choices in the trade-off between motor control and treatment adverse effects. Considering that levodopa is more effective and safer than the other dopaminergic drugs, it should be the preferred treatment for motor symptoms at this stage^25^. Reinforcing the recommendation to take levodopa away from meals can help reduce delayed ONs. Soluble levodopa tablets, which are absorbed faster, are also useful to improve delayed ON^37^. Reduction of OFF periods can be achieved by increasing levodopa dose or adding catechol-O-methyltransferase inhibitors (iCOMT), however both strategies can lead to supratherapeutic levels of levodopa, contributing to adverse effects and peak-dose dyskinesias^25^ ^,^ ^38^.

Controlled release formulations of levodopa have longer bioavailability and could theoretically improve ON time, however it is still unclear whether these are helpful in controlling motor fluctuations. Night time rigidity and akinesia, on the other hand, could be improved by a bedtime dose of controlled release levodopa.16 Sudden OFF periods and morning akinesia could be improved by rescue apomorphine injections^39^. 

## TREATMENT OF LEVODOPA-INDUCED DYSKINESIAS (LID)

Correct identification of the type of dyskinesias is of paramount importance for treatment success. Diphasic and dystonic dyskinesias could be improved by increasing levodopa dosage, whereas peak-dose dyskinesias usually respond to a reduction in levodopa dose or the total amount of dopamine replacement therapy^25^. When treating peak-dose dyskinesias it is important to consider that most patients are not bothered by the involuntary movements nor perceive any functional impairment related to peak dose dyskinesias. A reduction of levodopa dosage is not always necessary and could lead to motor deterioration^40^. 

When levodopa dose reduction with the goal of ameliorating dyskinesias results in inadequate motor control, add-on therapy, with dopamine agonists or MAOi, is usually recommended to patients in earlier stages of PD. However, due to potential side effects, these medications are not recommended for individuals with advanced disease. Controlled release levodopa has not been shown to be superior to standard release levodopa in controlling dyskinesias^35^ ^,^ ^37^. 

Amantadine is the only medication that acutely improves peak-dose dyskinesias without worsening motor symptoms. The initial dose is 100 mg daily, which can be increased up to 400 mg daily, depending on tolerability. In advanced PD amantadine should be used with caution, because of higher propensity to side effects, particularly visual hallucinations and executive dysfunction. In the presence of cognitive impairment, an alternative treatment should be sought^35^.

Safinamide, a drug with dopaminergic and anti-glutamatergic properties appears to improve dyskinesias in a long-term scenario at a dose of 100 mg daily, but it appears to show no acute effect in reducing the severity of peak dose dyskinesias^41^. Clozapine, an atypical antipsychotic drug, can improve peak-dose dyskinesias. However, the need to monitor white blood cells count and cardiovascular risk makes it an unsuitable option for most patients^35^.

## INFUSION THERAPIES

Infusion therapies can deliver levodopa to the brain in a more constant way, bypassing the gastrointestinal tract and minimizing absorption rate variability. Potential benefits are reduction of OFF periods and adverse events associated with suprathreshold levels of levodopa, such as peak-dose dyskinesias, making these therapies suitable for individuals with advanced PD. Two types of infusion therapies are available, levodopa-carbidopa intestinal gel (LCIG) and apomorphine continuous infusion. 

LCIG is administered continuously via a percutaneous endoscopic gastrostomy-jejunostomy feeding tube and has the inconvenience of relying on an infusion system^34^. Apomorphine is a short-acting dopamine agonist with pharmacological properties akin to levodopa, with higher affinity for dopaminergic D1 and D2 receptors, that can also be administered via subcutaneous continuous infusion. Both LCIG and apomorphine infusion are effective in reducing OFF time in individuals with advanced PD and motor complications^42^ ^,^ ^43^ making them good therapeutic options for individuals who are not candidates for deep brain stimulation. These drugs are safe for individuals with a present or past history of impulse control behaviours^44^ ^,^ ^45^. 

## SURGERY

Many of the complications of advanced stage PD, such as postural instability, falls and cognitive impairment, make individuals at this stage of the disease unsuitable for deep brain stimulation (DBS)^1^. In a recent systematic review, DBS was superior to best medical treatment and similar to LCIG in controlling motor symptoms of advanced PD. In suitable patients the decision to undergo DBS should be individualized, considering risks, potential benefits, costs and life expectancy^46^. Lesioning procedures can also be considered if surgical risk permits. Pallidotomy, for instance, is effective in reducing levodopa-induced dyskinesias and OFF period disability^47^ ^,^ ^48^. 

## TREATMENT OF NONMOTOR SYMPTOMS

Comprehensive treatment of nonmotor symptoms is beyond the scope of this section, which will focus on the NMS more frequently seen in advanced PD. NMS are a significant source of distress and have a negative impact on the quality of life of individuals with PD, particularly in advanced disease stages^22^. NMS can e be a consequence of the disease or a complication of DRT. When NMS occurs in association with OFF symptoms (nonmotor OFF), NMS could be improved using the same strategies used to minimize motor OFF periods^22^ ^,^ ^25^. The most common nonmotor OFF symptoms are pain, akathisia, depression, anxiety, dysphoria and dysautonomia^22^.

Considering that many NMS are worsened by DRT and/or difficult to distinguish from complications of treatment, individuals with advanced PD and significant NMS benefit from dose reduction and simplification of treatment regimen (favoring levodopa over other classes of medication). This is particularly true for individuals with psychosis or cognitive impairment^25^. If cognitive impairment remains an issue in patients on levodopa monotherapy, the treating physician should attempt to establish the minimum levodopa dose necessary to control motor symptoms. Cholinesterase inhibitors could be prescribed, if motor deterioration prevents further reduction of levodopa in individuals with significant cognitive impairment. Rivastigmine and donepezil have both been shown to improve cognitive function and psychosis in PD^49^. If psychosis in advanced PD fails to improve with the measures discussed above, antipsychotics can be prescribed. Quetiapine and clozapine are the only antipsychotics that do not worsen parkinsonian symptoms, and are both useful to treat psychosis and visual hallucinations^25^. 

Orthostatic hypotension is common in advanced PD. Reduction of levodopa and other contributing medication should be attempted, if possible, in addition to non-pharmacological measures, such as increased fluid and salt intake, avoiding carbohydrate rich meals, and preferring smaller, more frequent meals. There is still no consensus guiding the decision to initiate pharmacological treatment. The drugs available to treat orthostatic hypotension are domperidone, fludrocortisone, droxidopa and midodrine^50^.

Impulse control behaviors (ICB) mainly affect individuals with young onset PD, but a significant proportion of patients remain symptomatic in thr long run ^51^. Reduction of DRT and/or initiation of infusion therapies are treatment options for individuals with advanced PD and troublesome ICBs^52^.

## PALLIATIVE CARE

Palliative care in PD focuses on improving quality of life and autonomy, planning advance care, and supporting caregivers. In advanced PD it should be seen as a complementary treatment strategy that could be used in parallel to other treatments. Decision to start palliation is difficult considering the variability in clinical phenotypes. Generally, limited lifetime expectancy, rapid disease progression, malnutrition, impaired breathing, or the occurrence of life-threatening complications in the previous year are clues that could be used to identify individuals who would benefit from palliative care. Palliative care can also be indicated with the prescription of a new intervention or when end of life is approaching^53^.

## PARKINSON DISEASE REHABILITATION

Although pharmaceutical treatments improve PD manifestations, optimal management requires a multidisciplinary approach. Independently of the stage of the disease, patients and caregivers may benefit from tailored rehabilitation programs according to their needs^54^. Many rehabilitation interventions have been proven to improve PD symptoms^55^ and clinical practice guidelines have been published to help therapists and neurologists to provide best decision making for the management of PD patients treatment^56^.

A recent meta-analysis evaluated the effect of different physical therapy (PT) interventions on motor symptoms, balance, gait and quality of life (QoL)^57^. The authors concluded that conventional PT improved motor symptoms, gait, and QoL. Resistance and treadmill training improved gait, hydrotherapy improved balance, and strategy training improved balance and gait. Dance, Nordic walking, martial arts, and balance and gait training improved motor symptoms, balance, and gait. Dual task training (DT) did not improve motor symptoms, balance, gait and QoL. Although this last finding might seem conflicting with proven benefits of DT ^55^ ^,^ ^58^ ^,^ ^59^, DTT should be implemented in PD rehabilitation, because of the motor learning principle^60^. Patients improve mobility and cognitive function when both motor and cognitive activities are integrated during DT training^55^. Caution should be taken with people with higher falling risk, freezing of gait or mild cognitive impairment,. Good strategies and tailoring exercises to each patient’s personal and physical cognitive abilities^61^ may reduce or avoid risks during training.

 Many studies suggest that aerobic and high intensity exercises may attenuate PD symptoms^62^. Resistance training improves muscle strength, mobility, balance, functional capacity, QoL and reduces the risk of falls of PD patients^63^. Exercise may improve cognitive functions of PD patients at a mild to moderate stage of the disease^64^. Exergaming and virtual reality training can be useful to improve gait in PD^65^. Whether these effects are merely symptomatic or reflective of a possible disease-modifying effect requires further study^66^. Overall, there are long-term effects with exercise training^67^ but there is no consensus about which types of exercises are better for PD, and, therefore, individualized prescription, similarly to pharmacotherapeutic interventions, such as frequency, duration, and intensity, is required^68^. 

Recent meta-analysis demonstrated the efficacy of the Lee Silverman Voice Treatment in increasing vocal loudness and functional communication among individuals with PD^69^. Speech language therapy (SLT) has potential benefits for PD dysphagia^70^. Intensive SLT is effective for hypophonia and can lead to some improvement of voice pitch but behavioral speech rehabilitation in PD still needs validation^71^.

Although occupational therapy (OT) has a perceived possibly beneficial role for PD, studies about the effect of OT in PD are scarce. In 2014, Sturkenboom et al.^72^ demonstrated that OT can improve the self-perceived performance of PD patients in daily activities. Different OT interventions may be effective in improving QoL in patients with PD. Because of the lack of high-quality studies, further investigations are needed to establish firm conclusions about OT efficacy in PD^73^.

## AN ALGORITHM FOR THE TREATMENT OF MOTOR SYMPTOMS OF PD AND RECENT GUIDELINES FOR THE TREATMENT OF PARKINSON’S DISEASE

**Figure 1.**
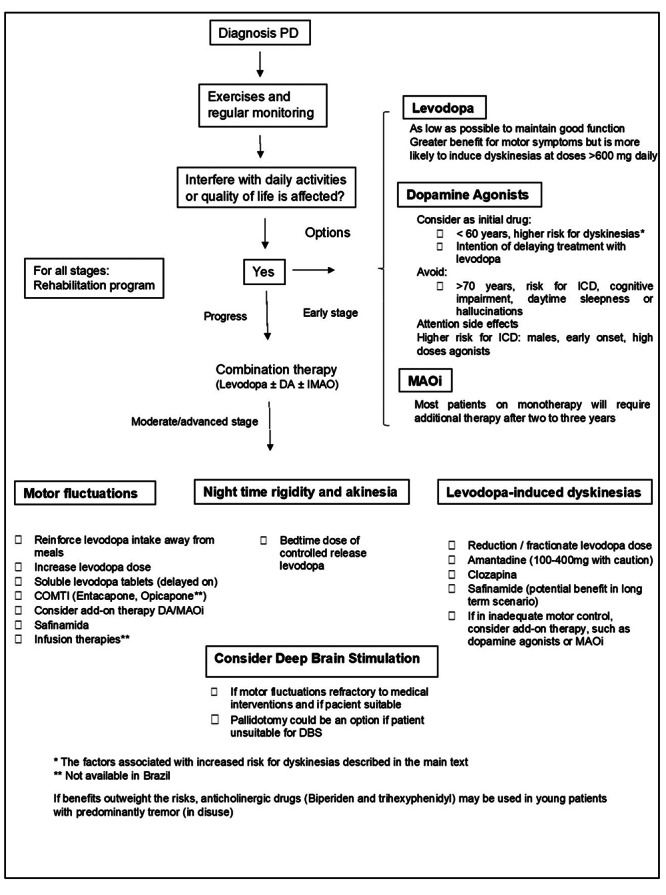
Treatment of PD motor symptoms.

Based on this review, we propose an algorithm as a guide for the treatment of motor symptoms in PD Figure 1, Moreover, it is important to mention three recently published guidelines with recommendations concerning the treatment of PD: the Canadian guideline^4^, the Brazilian guideline of the Movement Disorders Scientific Department of the Brazilian Academy of Neurology^74^ and the American Academy of Neurology guideline^75^. They review the current evidence of available treatment options for PD. Additionally, an update on the management of PD for general neurologists was recently published by Pyrtosek et al^76^. These written materials can help doctors to improve treatment strategies for PD patients.
